# SNARE-dependent glutamate release in megakaryocytes

**DOI:** 10.1016/j.exphem.2010.03.011

**Published:** 2010-06

**Authors:** Catherine J. Thompson, Tatjana Schilling, Martin R. Howard, Paul G. Genever

**Affiliations:** a Department of Biology, University of York, York, United Kingdom; b Department of Haematology, York Health Services, National Health Service Trust, York, United Kingdom

## Abstract

**Objective:**

The identification of signaling pathways involved in megakaryocytopoiesis is essential for development of novel therapeutics to treat hematological disorders. Following our previous findings that megakaryocytes express functional channel-forming *N*-methyl-D-aspartate-type glutamate receptors, here we aimed to determine the glutamate release capacity in undifferentiated and differentiated megakaryocytes and the role of soluble N-ethyl maleimide-sensitive factor attachment protein receptor (SNARE) proteins that are known to be associated with vesicular exocytosis.

**Materials and Methods:**

Using the megakaryocytic cell line MEG-01, primary megakaryocytes, and tissue sections of bone marrow, reverse transcription polymerase chain reaction, Western blot analysis, and immunolocalization were employed to detect factors required for vesicular glutamate release. Vesicle recycling was monitored by acridine orange and FM1-43 staining and glutamate release activity was assessed by an enzyme-linked fluorimetric assay. Genetically modified MEG-01 cells, with deletion or overexpression of SNARE and vesicular proteins, were also examined for glutamate release activity.

**Results:**

We demonstrated that megakaryocytes express numerous proteins required for vesicular glutamate release, including core SNARE proteins, vesicle-associated membrane protein, soluble N-ethyl maleimide-sensitive factor attachment protein−23, and syntaxin, as well as specific glutamate-loading vesicle proteins, VGLUT1 and VGLUT2. Moreover, active vesicle recycling and differentiation-dependent glutamate release were observed in megakaryocytes. Vesicle-associated membrane protein−deficient MEG-01 cells, which are impaired in vesicle recycling, showed a 30% decrease in released glutamate, whereas overexpression of VGLUT1 exhibited up to a 2.2-fold increase in glutamate release.

**Conclusion:**

These data show that glutamate release from megakaryocytes occurs in a SNARE-dependent, exocytotic manner and is increased during differentiation, suggesting that manipulation of glutamate signaling could influence megakaryocytopoiesis and, therefore, offer a suitable target for the treatment of thrombosis and other hematological disorders.

Megakaryocytes (MKs) are required for production and release of platelets (thrombopoiesis). In order to maintain normal hemostasis, i.e., adequate numbers of platelets circulating in peripheral blood, the c-Mpl ligand thrombopoietin (TPO) acts as the major hormone controlling megakaryocytopoiesis [1,2]. Numerous additional cytokines, such as stem cell factor, interleukin-1(IL-1), IL-3, IL-6, IL-11, and granulocyte colony-stimulating factor, are also involved [3,4]. However, the exact cell signaling pathways involved in this process are still to be determined. In addition, there are at least four recognized TPO-independent signaling mechanisms that regulate megakaryocytopoiesis: gp130-dependent pathways, Notch, stromal-derived factor/fibroblast growth factor 4, and *N*-methyl-D-aspartate (NMDA)-type glutamate signaling [5].

Glutamate signaling was once thought to be restricted to the mammalian central nervous system (CNS), where it is involved in aspects of normal brain function, including learning, cognition, and memory [6,7]. However, glutamate signaling networks have been identified in non-neuronal tissues, such as bone, spleen, lung, liver, pancreas, heart, stomach, testis, and intestine [8,9]. We have previously reported that primary human MKs, rat bone marrow MKs, and the MEG-01 megakaryoblastic cell line express functional channel-forming NMDA-type glutamate receptors, and that antagonism of the NMDA receptor by MK-801 inhibited normal MK development and platelet production [10,11]. Glutamate transporters, which are responsible for glutamate uptake, have been identified on platelets [12,13], and it has been demonstrated that functional α-amino-3-hydroxy-5-methyl-4-isoxazole proprionic acid−type glutamate receptors are expressed by platelets and are required for platelet activation [14]. Together, these findings indicate that glutamate signaling has a fundamental role in MK development as well as platelet production and function. However, the local cellular source of the glutamate agonist and the mechanisms that regulate its release are not known. We hypothesized that MKs were also able to send glutamate signals using exocytotic mechanisms similar to presynaptic neurons.

Membrane trafficking in presynaptic nerve terminals in the CNS is essential for glutamate secretion and involves biogenesis of specialized vesicles that perform a regulated cycle of fusion, exocytosis, and regeneration [15,16]. Exocytosis of neurotransmitter is a complex and highly regulated process involving specific protein−protein interactions. The current model is the soluble N-ethyl maleimide-sensitive factor attachment protein (SNAP) receptor (SNARE) hypothesis, which proposes that specific vSNARE proteins on the vesicle membrane recognize and interact with the target protein tSNAREs on the plasma membrane of presynaptic cells [17]. This membrane protein complex is composed of vesicle-associated membrane protein (VAMP) alias synaptobrevin (vSNARE) and the tSNARE proteins syntaxin and SNAP-25 (synaptosomal-associated protein, 25 kDa). These proteins interact with each other in a structurally conserved parallel four-helix bundle, which brings the vesicle and plasma membranes into close contact so that they are ready to fuse [18–20]. There are numerous SNARE-interacting proteins that regulate SNARE complex assembly and disassembly. SNARE assembly is triggered by the binding of calcium to a putative calcium sensor, synaptotagmin. This induces the fusion of synaptic vesicles docked at the active zone close to calcium channels. Following membrane fusion, disassembly of the SNARE complex is regulated by two proteins, the ATPase N-ethyl maleimide-sensitive factor (NSF) and an adapter protein α-SNAP (soluble NSF attachment protein). SNARE complex formation is also regulated by syntaxin-interacting proteins, such as MUNC-18; guanosine triphosphate−binding protein Rab 3, and Rab 3−binding proteins Rabphilin 3, which are involved in exocytosis via hydrolysis of guanosine triphosphate, and vesicular adapter proteins Doc-2 and Mint, which regulate MUNC-18/syntaxin interactions [21–25]. Specific transporters expressed on the vesicle membrane determine the transmitter cargo that will be packaged, carried, and subsequently released following fusion with the plasma membrane. The vesicular glutamate transporters (VGLUTs) are markers of presynaptic glutamatergic neurons and were thought to be restricted to the CNS, where they function to load glutamate into recycling intracellular vesicles [26–28].

In this study, we demonstrate that MKs express a range of functional SNARE and related proteins required for vesicular glutamate exocytosis confirming glutamatergic signaling activity in MKs, which is further increased after differentiation.

## Materials and methods

Plasticware and reagents used for cell culture were purchased from Life Technologies (Paisley, UK) unless otherwise stated. Cells were maintained in a humidified atmosphere at 37°C in 5% CO_2_. All cell culture media were supplemented with 100 U/mL penicillin, 100 μg/mL streptomycin, and 2 mM l-glutamine, unless stated otherwise. All other chemicals were purchased from Sigma (Dorset, UK), unless stated otherwise.

### Cell culture

The MEG-01 megakaryoblastic cell line was maintained in suspension culture in RPMI-1640 medium and 10% fetal bovine serum and replated at 1 × 10^5^ cells/mL with each passaging. Megakaryocytic differentiation was induced by addition of 100 nM phorbol myristate acetate (PMA) for 72 hours.

### Murine and human megakaryocytes

Primary murine MKs (mMKs) were isolated from B6Cba mice as described previously [29], with minor modifications. Briefly, bone marrow was flushed out of femora and tibiae with Iscove's modified Dulbecco's medium (Sigma) containing 2% fetal bovine serum. The suspension was passed through a needle and sieved through a 100-μm filter. The cell pellet was resuspended in Opti-MEM supplemented with 5% baby hamster kidney cell TPO-conditioned media (baby hamster kidney TPO cells engineered to constitutively release TPO were a kind gift from Prof. Kenneth Kaushansky, UCSD, San Diego, CA, USA) [30]. Megakaryocytes were purified after 3 days on a gradient of bovine serum albumin.

Primary human MKs (hMKs) were generated from hematopoietic progenitor cells in umbilical cord blood obtained with informed consent as described previously [11]. Briefly, CD34^+^ cells were isolated by magnetic immunoselection (MACS; Miltenyi Biotec, Surrey, UK) and cultured in Iscove's modified Dulbecco's medium supplemented with 0.2% (w/v) bovine serum albumin, 1 mM sodium pyruvate, 1% minimum essential medium nonessential amino acids, 1% minimum essential medium vitamins, 0.1 mM 2-mercaptoethanol, 10% cord blood plasma, and 40 ng/mL TPO (R&D Systems, Abingdon, UK). Cells were plated at 1.5 × 10^5^ cells/mL, replated after 7 days and cultured for another 7 days.

### Transfected MEG-01 cells

Active tetanus toxin light chain contained in the expression construct pTNT (a kind gift from Dr. S. Sweeney, University of York, UK [31]) was directionally cloned into pcDNA3.1/V5-HIS B (Invitrogen, Carlsbad, CA, USA). Primers used to generate the VGLUT1 constructs were: 5′-ttagcagatctcaggagccgccaccat-3′ and 5′-gattacgtcgacgggaggcacatggtctgtag-3′ with a 1 base-pair mismatch in the reverse primer to allow for fusion with enhanced green fluorescent protein (EGFP) encoded in pEGFP-N2 (Clontech, Saint-Germain-en-Laye, France) after being directionally cloned into this vector. VGLUT1 was also directionally cloned into pcDNA3.1/V5-HIS B using the following primers: 5′-ttagcagatctacaggagccgccaccat-3′ and 5′-gattacggtaccgggaggcacatggtcagtag-3′. MEG-01 cells were transiently transfected using Lipofectamine 2000 reagent (Invitrogen) according to the manufacturer's instructions. The pGL3-luciferase plasmid (Promega, Southampton, UK) was used to determine transfection efficiency.

### Immunolocalizations

Cryosections of tibiae and femora from neonatal rats were fixed in 4% paraformaldehyde and exposed to 3% hydrogen peroxide to deplete endogenous peroxidase activity. After blocking with appropriate serum, sections were incubated with primary antibodies (anti-VGLUT1, anti-VGLUT2, anti−SNAP-23, all Synaptic Systems, Coventry, UK; anti−syntaxin 4 and anti-CD61, both BD Biosciences, Oxford, UK) followed by corresponding biotinylated secondary antibodies (Vector Laboratories, Peterborough, UK). Sections were then exposed to an avidin-biotinylated peroxidase reagent (ABC Elite; Vector Laboratories) prior to visualization of peroxidase activity using 3,3′-diaminobenzidine. Sections were counterstained with Mayer's hematoxylin before mounting in glycerol/phosphate-buffered saline. Negative controls received the same concentration of an appropriate immunoglobulin G (Vector Laboratories) in place of primary antibody.

For immunofluorescent localization of SNARE complex and associated proteins in MEG-01 cells and mMKs, cytospins were fixed in 4% paraformaldehyde, blocked with appropriate serum, and incubated with primary antibodies (anti-VGLUT1, anti-VGLUT2, anti−SNAP-23, anti-synaptotagmin, all Synaptic Systems; anti−syntaxin 4, BD Biosciences; and anti-VAMP1/2, Sigma). Antibody binding was determined using corresponding fluorescein isothiocyanate−conjugated secondary antibodies (anti-rabbit fluorescein isothiocyanate; Santa Cruz, Heidelberg, Germany; anti-mouse fluorescein isothiocyanate; Sigma). Samples were mounted in Vectashield mounting medium containing 4′,6-diamidino-2-phenylindole (Vector Laboratories) and imaging was performed using a confocal laser scanning microscope (Carl Zeiss, Welwyn Garden City, UK).

### Western blot analysis

MEG-01 cells, primary hMKs, and human brain homogenates (Abcam, Cambridge, UK) were lysed using 20 mM Tris (pH 7.5), 150 mM NaCl, 1 mM ethylene diamine tetraacetic acid, 1 mM ethylene glycol tetraacetic acid, 1% Triton X-100, 2.5 mM sodium pyrophosphate, 1 mM β-glycerol phosphate, 1 mM NaVO_4_, and 1 μg/mL leupeptin, separated by sodium dodecyl sulfate polyacrylamide gel electrophoresis and transferred onto a nitrocellulose membrane. After blocking with 4% skim milk powder in TBS-T (20 mM Tris, 137 mM NaCl, 0.1% Tween 20), membranes were incubated with primary antibodies (anti-VAMP1/2; Sigma, anti−SNAP-23; Synaptic Systems, anti−syntaxin 4; BD Biosciences, anti−glyceraldehyde phosphate dehydrogenase; Advanced ImmunoChemical, Long Beach, CA, USA). After washing and incubation with the corresponding horseradish peroxidase−conjugated secondary antibodies (Santa Cruz), specific antibody binding was detected using enhanced chemiluminescent solution and Hyperfilm (GE Healthcare, Little Chalfont, UK).

### Reverse transcriptase-polymerase chain reaction (PCR)

Total RNA was extracted from MEG-01 cells, hMKs, and mMKs using TRIzol reagent (Invitrogen). Complementary DNA was synthesized using SuperScript II reverse transcriptase (Invitrogen) and used for PCR with gene-specific primers (Sigma; Table 1). PCR was performed for 35 cycles of 94°C for 10 seconds, annealing temperature for 20 seconds, and 72°C for 1 minute. PCR products were sequenced to confirm their specificity.

### Acridine orange and FM1-43 staining

Acidic vesicles were stained with 5 μg/mL acridine orange in culture medium. After 15 minutes at 37°C, the cells were pelleted and washed with phosphate-buffered saline prior to imaging.

Vesicle recycling was determined by FM1-43 staining as described previously [32]. Briefly, MEG-01 cells and mMKs were plated at 5 × 10^4^ cells/cm^2^ onto coverslips and cultivated for 24 hours. Cells were washed with Hank's buffered saline solution (HBSS) containing 1 mM CaCl_2_. Recycling vesicles were labeled using 10 μM FM1-43 (Invitrogen) for up to 20 minutes. Cells were washed in HBSS and fixed in 4% paraformaldehyde prior to imaging. For destaining experiments, MEG-01 cells were stained with FM1-43 for 20 minutes, washed in HBSS and incubated for 30 to 60 minutes in FM1-43-free HBSS.

### Glutamate release assay

Glutamate release was determined using an enzyme-linked fluorimetric assay based on previously published protocols [33] with some modifications [32]. Briefly, cells were plated at 5 × 10^5^ cells/mL in blackened 96-well plates (Greiner, Stonehouse, UK) in release buffer, containing 120 mM NaCl, 3 mM KCl, 1.25 mM NaH_2_PO_4_, 25 mM HEPES-Na, 4 mM glucose, 1 mM MgCl_2_, and 2 mM CaCl_2_ (pH 7.4). The reaction was initiated after 30 minutes by the addition of 40 U/mL glutamate dehydrogenase and 1 mM NADP^+^. Fluorescence for samples and standards of known concentrations of exogenous glutamate were monitored using a fluorescence plate reader (Dynex Technologies, Worthing, UK) with a thermostated incubation chamber (37°C). To determine intracellular glutamate concentrations, cells were incubated with 0.1% Triton X-100 before performing the assay. Glutamate release was normalized to cell number and statistically analyzed by one-way analysis of variance.

## Results

### Expression of SNARE complex and accessory proteins in megakaryocytes

**Figure 1:**
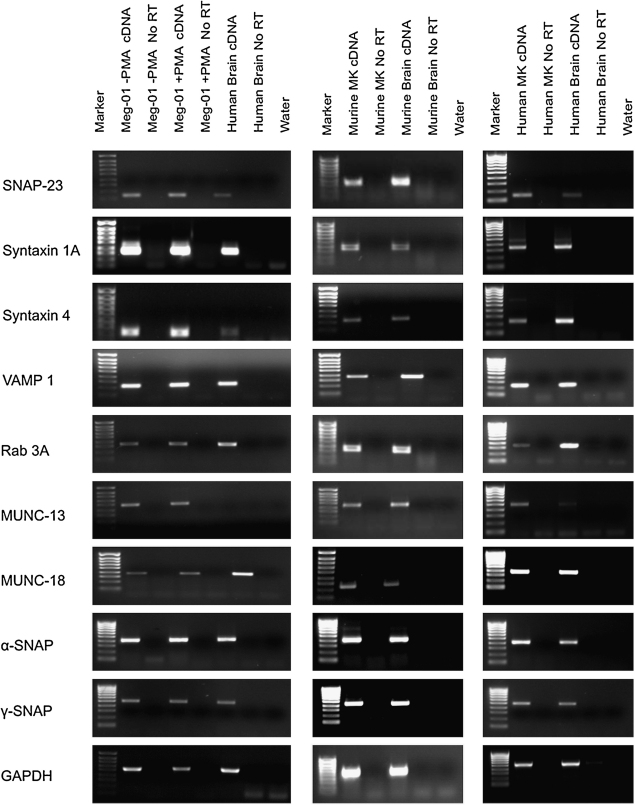
Expression of soluble N-ethyl maleimide-sensitive factor attachment protein receptor (SNARE) complex and accessory genes by megakaryocytes. Reverse transcription polymerase chain reaction was used to determine messenger RNA expression of SNARE complex and accessory proteins in MEG-01 cells (left panel), primary murine megakaryocytes (MKs, middle panel) and primary human MKs (right panel). For MEG-01 cells, expression in the undifferentiated (−phorbol myristate acetate [PMA]), as well as differentiated (+PMA) state is shown. Human fetal forebrain or mouse whole brain was used as a positive control. Negative controls (no reverse transcriptase [RT] and water) were performed in parallel.

**Figure 2:**
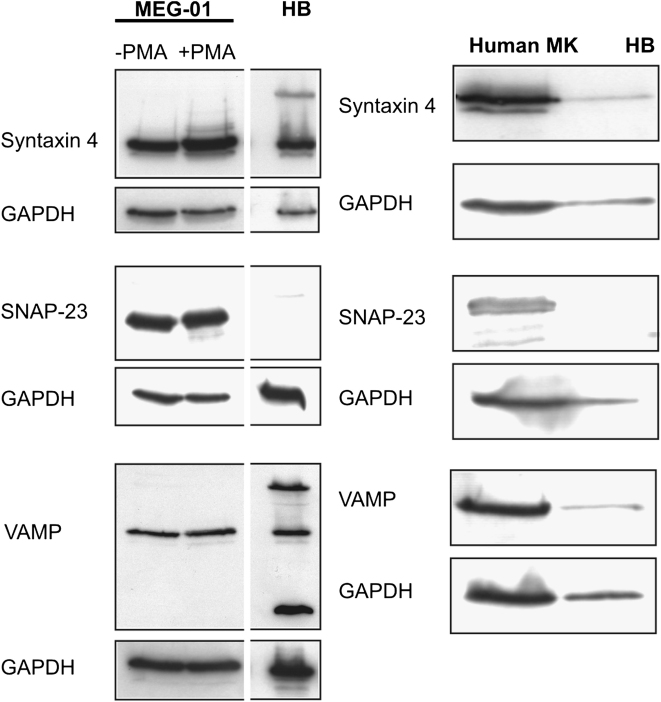
Western blot analysis of soluble N-ethyl maleimide-sensitive factor attachment protein receptor (SNARE) complex proteins in megakaryocytes. Expression of SNARE complex proteins SNAP-23, Syntaxin 4, and vesicle-associated membrane protein (VAMP) was determined by Western blot analysis in MEG-01 cells, in the undifferentiated (−phorbol myristate acetate [PMA]), as well as differentiated (+PMA) state, and in primary human megakaryocytes (MK). Human brain (HB) lysate was used as a positive control, detection of glyceraldehyde phosphate dehydrogenase (GAPDH) was performed as loading control.

**Figure 3:**
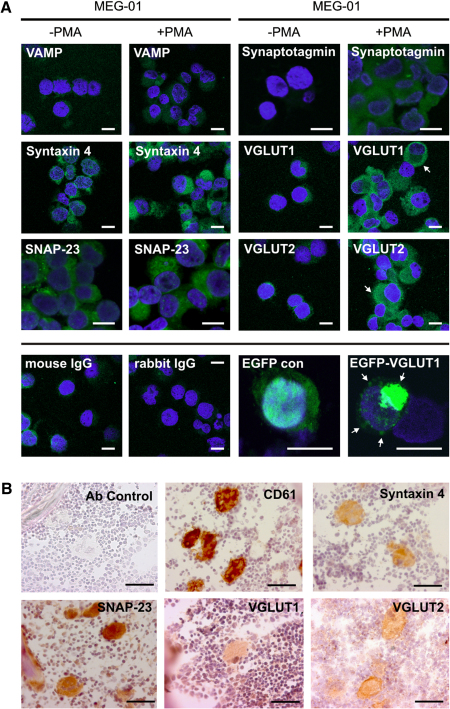
Immunolocalization of soluble N-ethyl maleimide-sensitive factor attachment protein receptor (SNARE) complex and accessory proteins in megakaryocytes. (**A**) Immunofluorescent localization of SNARE complex and accessory proteins (green) was determined by confocal laser scanning microscopy in MEG-01 cells in the undifferentiated (−phorbol myristate acetate [PMA]) as well as differentiated (+PMA) state, nuclei were stained with 4′,6-diamidino-2-phenylindole (blue); the bottom row shows negative controls for vesicle-associated membrane protein (VAMP) and Syntaxin (mouse immunoglobulin G) and for Synaptotagmin, SNAP-23, vesicular glutamate transporter (VGLUT) 1, and VGLUT2 (rabbit immunoglobulin G) immunostainings, as well as the overexpression of enhanced green fluorescent protein (EGFP)-VGLUT1 in undifferentiated (−PMA) MEG-01 cells and empty vector control (EGFP con); arrows indicate elevated accumulation of VGLUT1 and VGLUT2 at the cell periphery in differentiated MEG-01 cells as well as dense distribution of VGLUT1 in specific regions near the plasma membrane of VGLUT1-transfected cells; bar = 20 μm. (**B**) Brown staining reports expression of SNARE complex proteins (Syntaxin 4 and SNAP-23) and VGLUT1/2, respectively, in rat bone marrow. Detection of CD61 was used to confirm megakaryocyte identity. Nuclei were counterstained with hematoxylin (blue); bar = 30 μm.

MEG-01 cells, mMKs, and hMKs expressed SNARE complex and essential accessory components on the gene transcription and protein level (Figs. 1–3). Messenger RNA expression of core complex proteins VAMP1 and syntaxin 4 were identified in MEG-01 cells treated with and without PMA and in primary MKs (Fig. 1). Expression of SNAP-25 was not found but its homologue SNAP-23 was identified. The accessory proteins NSF, Rab 3A, MUNC-13, MUNC-18, α-SNAP, and γ-SNAP were expressed in MEG-01 cells treated with and without PMA, and in primary MKs. SNAP-23, VAMP, and syntaxin 4 expression was confirmed by Western blot analysis (Fig. 2) in MEG-01 cells, treated with and without PMA, and hMKs. SNARE and accessory proteins were also identified at the protein level in MEG-01 cells and primary MKs using immunofluorescent localization (Fig. 3A) and immunohistochemistry (Fig. 3B). Cytoplasmic VGLUT1 and VGLUT2 were identified in MEG-01 cells and rat bone marrow MKs, with occasional dense distribution at the cell periphery. Similarly, in MEG-01 cells overexpressing EGFP-tagged VGLUT1, the localization was preferentially located in specific regions near the plasma membrane, compared to generalized, diffuse distribution in EGFP-only controls (Fig. 3A).

### Identification of acidic vesicles in megakaryocytes

**Figure 4:**
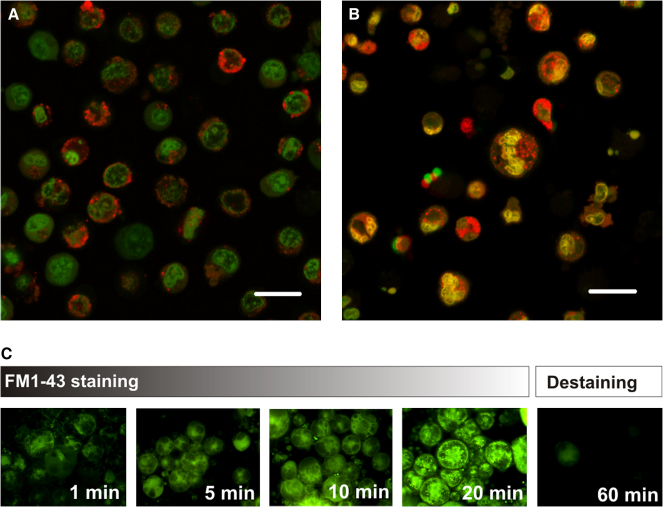
Identification of acidic, recycling vesicles in megakaryocytes. (**A**) After acridine orange staining, acidic vesicles were observed in the MEG-01 cells (red) in the periphery of the cells. Green fluorescence reports soluble dye within the cytosol. (**B**) Acidic vesicles were also observed in primary human megakaryocytes (MKs), where additionally to their localization at the periphery of the cells (red), they colocalized with soluble dye in the cytosol of the cell (yellow); bars = 20 μm. (**C**) Vesicular recycling in primary murine MKs was detected by incorporation of the FM1-43 dye into vesicle membranes during endocytosis, which leads to an increase of green fluorescence within the cells. Following FM1-43 removal, the cells destained within 60 minutes reporting exocytosis of these vesicles.

Acidic, synaptic-like vesicles were identified in MKs using the cell-permeable, pH-sensitive dye acridine orange. After being trapped in acidic vesicles, acridine orange exhibits red emission, whereas green fluorescence occurs in its soluble form or when bound to double-stranded DNA [34,35]. In MEG-01 cells, red fluorescence was observed around the periphery of the cells, and green fluorescence within the cytosol (Fig. 4A). Acidic vesicles were also detected in hMK cells, where green and red fluorescence colocalized in the cytosol, while red fluorescence alone was observed in the periphery of the cells (Fig. 4B).

### Identification of vesicular recycling in megakaryocytes

Vesicle recycling activity was shown in mMKs by FM1-43 staining. FM1-43 integrates into the plasma membrane of cells, is incorporated into the vesicle membrane as vesicles fuse with the plasma membrane and acts as a fluorescent reporter of vesicle recycling. The accumulative increase in the amount of fluorescent dye within the cells over a 20-minute incubation period corresponded to the uptake of vesicles (Fig. 4C). The loss of fluorescence following removal of FM1-43 reported exocytosis of these vesicles.

### Glutamate release by megakaryocytes and its regulation by the SNARE complex

**Figure 5:**
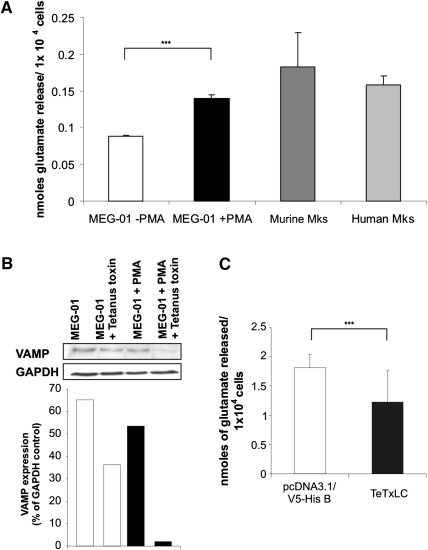

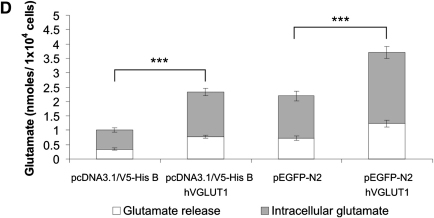
Glutamate release in megakaryocytes. (**A**) After 72 hours of cultivation, an enzyme-linked fluorimetric assay was used to detect glutamate release from MEG-01 cells in the undifferentiated (−phorbol myristate acetate [PMA]) as well as differentiated (+PMA) state and from primary murine and human megakaryocytes (MKs) (n = 3, mean + standard deviation; ^∗∗∗^*p* < 0.001). (**B**) Western blot analysis (evaluated by densitometry normalized to the housekeeping protein glyceraldehyde phosphate dehydrogenase [GAPDH]) demonstrated that treatment with 100 nM tetanus toxin decreased vesicle-associated membrane protein (VAMP) protein levels in MEG-01 cells in the undifferentiated (−PMA; 45% decrease compared to undifferentiated control) as well as differentiated (+PMA; 94% decrease compared to differentiated control) state, which inhibits formation of the SNARE complex. (**C**) Using the enzyme-linked fluorimetric assay with MEG-01 cells overexpressing the tetanus toxin light chain (TeTxLC) demonstrated a 30% reduced glutamate release as a result of the inhibition of the SNARE complex due to reduction of VAMP compared to empty vector control (pcDNA3.1/V5-His B; n = 3, mean + standard deviation; ^∗∗∗^*p* < 0.001). (**D**) Two different transfection vectors (pcDNA3.1/V5-HIS B and pEGFP-N2) were used to introduce VGLUT1 overexpression into MEG-01 cells. Glutamate released from VGLUT-overexpressing cells, as well as intracellular glutamate was detected by an enzyme-linked fluorimetric assay. Compared to empty vector controls (pcDNA3.1/V5-His B and pEGFP-N2, respectively), glutamate release was 2.26- and 1.69-fold increased and intracellular glutamate was 2.29- and 1.69-fold elevated in pcDNA3.1/V-His B hVGLUT1- and pEGFP-N2 VGLUT1-transfected cells, respectively (n = 3, mean + SD; ^∗∗∗^*p* < 0.001).

MEG-01 cells and primary MKs spontaneously released glutamate, which was significantly enhanced in differentiated MEG-01 cells compared to cells without PMA treatment (Fig. 5A). To determine whether the SNARE complex is responsible for glutamate release from MKs, depletion and overexpression of SNARE-associated proteins were assessed. Firstly, the effect of tetanus toxin, which cleaves VAMP to disable vesicle recycling by preventing the formation of the SNARE complex [36], was examined. Treatment of MEG-01 with tetanus toxin decreased VAMP protein in MEG-01 cells in the absence and presence of PMA (Fig. 5B). Transient transfection of MEG-01 cells with tetanus toxin induced a 30% decrease (*p* < 0.001) in released glutamate (Fig. 5C). MEG-01 cells overexpressing the VGLUT1 protein showed a 2.26- and 1.69-fold increase (*p* < 0.001) of released glutamate and a 2.29- and 1.69-fold elevation (*p* < 0.001) of intracellular glutamate, respectively, when compared to the respective vector control (pcDNA3.1/V5-His B and pEGFP-N2) (Fig. 5D).

## Discussion

We have demonstrated that MKs have the ability to release glutamate in a manner similar to presynaptic neurons in the CNS involving SNARE-dependent exocytosis. Several components of the core exocytotic complex and accessory proteins are the same as those identified in the CNS as mediators for glutamate exocytosis [17,18,20,37]. Instead of SNAP-25, which is present in the SNARE complex in neuronal cells, expression of its homologue tSNARE SNAP-23, which is vital for membrane fusion and vesicle docking in non-neuronal tissues [38], has been found in MKs. SNARE complex factors were also identified on the protein level in MKs. Consequently, the detection of SNARE complex factors and accessory proteins not only in cell cultures but also in sections of bone marrow, i.e., in situ, supports our results obtained by using a MK cell line and primary MKs. As one of the core proteins of the SNARE complex, VAMP was detected by Western blotting at approximately 36 kDa in MKs corresponding to its homodimeric form, whereas additionally, a monomeric VAMP band (18 kDa) and a 68-kDa band, according to literature corresponding to VAMP/synaptophysin heterodimers, were identified in the human brain lysate control [22,39].

Expression of VGLUT1 and VGLUT2 in MKs suggests that these vesicular glutamate transporters play a role in MKs similar to that seen in the CNS, where they are required for the uptake of glutamate into synaptic vesicles in glutamate-releasing neurons [40]. We observed immunolocalization of endogenous VGLUT proteins in both primary MKs and MEG-01 cells, as well as localization of EGFP-tagged VGLUT1 in MEG-01 cells prominently at the plasma membrane consistent with vesicular release activity identified in peripheral specialized zones in neuronal cells [27]. Furthermore, overexpression of VGLUT1 in MEG-01 cells significantly increased the amount of glutamate released. Previous in vitro studies have detected increased glutamate release at synapses of VGLUT1-overexpressing glutamatergic neurons [41] and overexpression of *Drosophila* VGLUT in motoneurons in vivo has been reported to augment vesicular glutamate release, with an increase in synaptic vesicle volume and a decrease in the number of released vesicles to maintain normal levels of excitation at the synapses [42]. These data confirm the specificity of our findings and that glutamate signaling in MKs is at least in part VGLUT-dependent.

Endocytosis and exocytosis of acidic vesicles in MKs were monitored using the pH-sensitive dye acridine orange, as has been described for acidic neurotransmitter-containing vesicles in synaptosomes [43]. In neuronal cells, glutamate loading acidifies recycling synaptic vesicles and the simultaneous uptake of acridine orange results in red fluorescence. With exocytosis, this dye is released from synaptosomes, which decreases red fluorescence, while green fluorescence of the dye reports its non−vesicle-associated form. In both MEG-01 cells and hMKs, we identified abundant acidic vesicles in the cytosol with mainly peripheral localization in MEG-01 cells. Moreover, we employed FM1-43, a styrylpyridinium dye, to observe vesicle recycling, as has been shown for synaptosomal recycling in a range of species, including mouse, frog, and rat [44–47]. The incorporation of FM1-43 dye into MKs during a 20-minute time course indicates that vesicular endocytosis takes place within MKs. Destaining experiments, i.e., removal of the dye from the culture medium, caused a reduction of fluorescence, which provides evidence for vesicular exocytosis. Taking the results of both acridine orange and FM1-43 staining into account, they provide evidence that acidic neurotransmitter-containing vesicles are present in megakaryocytes and that they are participating in active vesicle recycling, as described for neuronal cells.

We next determined if megakaryocytes released glutamate in a SNARE-dependent manner similar to neurotransmitter release in synaptosomes [17,18,20,37]. Depletion of VAMP, which is mandatory for the formation of the SNARE complex [36], decreased glutamate exocytosis, whereas VGLUT overexpression increased SNARE glutamate release by stimulating the loading of glutamate into intracellular vesicles [26–28,48].

Furthermore, in mature megakaryocytes, which were obtained by differentiation of MEG-01 cells with PMA, VGLUT1 protein was more prominent in the differentiated state and glutamate release was significantly elevated. Recently, Isakari et al. [49] confirmed that treatment of MEG-01 cells with PMA can be used as a model of human megakaryopoiesis and platelet production. Their gene expression study also revealed elevated messenger RNA levels for SNAP-23 within 24 hours of differentiation. We observed SNAP-23 expression at the RNA, as well as on the protein level in both undifferentiated and differentiated MEG-01 cells after 72 hours of PMA treatment; however, we could not detect differential expression. This could be due to the different time points examined, but nevertheless confirms the activity of SNARE-associated factors during megakaryocytopoiesis.

The identification of key regulatory proteins, namely the putative calcium sensor synaptotagmin and Rab3A, in MKs provides evidence for Ca^2+^-dependent regulation of vesicular glutamate exocytosis in these cells. Via real-time glutamate-release monitoring, we could demonstrate that MKs released glutamate in the presence of Ca^2+^. However unlike neuronal cells, MKs are classified as nonexcitable cells and are not believed to express voltage-gated Ca^2+^ channels [50–52]. Voltage-gated Ca^2+^ channels enable rapid influx of Ca^2+^ as a fast response to an incoming stimulus, leading to neurotransmitter release. During this process, an intracellular linker that associates with synaptic proteins like synaptotagmin provides a tight interaction between presynaptic Ca^2+^ channels and the SNARE complex, which is essential for stabilizing neurotransmitter release [53]. Therefore, even though MKs express Ca^2+^-permeable NMDA glutamate receptor, our observations suggest that glutamate release mechanisms in MKs are different than those described for neurons; MKs rather seem to spontaneously release glutamate, which is increased during differentiation. This further implies that factors regulating the passive and active exocytosis of glutamate, respectively, play an important role during MK differentiation and should be addressed in future work.

In conclusion, our previous studies have shown that NMDA glutamate receptor signaling within MKs is important for megakaryocytopoiesis and platelet production in vitro [10,11]. Here, we provide strong evidence that SNARE-dependent glutamate-release mechanisms, similar to but distinct from those identified in the CNS, are present in MKs. The release of glutamate from MKs provides a cellular source of glutamate within the bone marrow compartment, which can potentially activate their NMDA glutamate receptors in an autocrine fashion controlling megakarocytopoiesis and platelet production. Moreover, we have previously shown that osteoblastic cells also express SNARE-associated and accessory proteins and are able to release glutamate [32,54]. This indicates that the osteoblastic cells provide an additional source of glutamate for the MKs and that both cell types may interact in the bone marrow. Together, these findings suggest that SNARE-dependent glutamate signaling plays a critical role in MK development and platelet production and may represent a route for novel therapeutic approaches to maintain normal hemostasis, for example, by generating anti-thrombotic agents.

## Acknowledgments

We thank Prof. Kenneth Kaushansky for kindly providing the baby hamster kidney TPO cells and for his advice on establishing the murine MK cultures. We also thank Dr. Sean Sweeney for supplying tetanus toxin construct and for helpful discussions. We are grateful to York Hospital (York, UK) for the provision of umbilical cord blood. This work was supported by a grant (FS/03/036/15438) from the British Heart Foundation (London, UK) to C.J.T.

## Conflict of Interest Disclosure

The authors declare no competing financial interests.

## Figures and Tables

**Table 1:** Primer sequences

Gene, species	Forward and reverse primer
α-SNAP, h/m	AGGCTCATCCAAAATAGAGGAAGCA, AGCCACCTTCAGCAGACACTTGTT
γ-SNAP, h	CACCTCGCCAAAGCAGAGAAATACC, AGTGCCGCCTCATCAAACCTACG
γ-SNAP, m	GATGCTGAAGGAGATGCAGAAGCTG, ACTGCCGTTAAAGCCTGGTATGCTG
GAPDH, h/m	GGTGAAGGTCGGWGTCAACGG, GGTCATGAGYCCTTCCACGAT
MUNC-13, h	AAGGCAGAACAGCAGGAGGA, GGACACTACATCACCCACAACC
MUNC-13, m	GCCTAAGTGTGGAGGTGTGGAACAA, GGCTGGGTAGTCGTGTGGTAAGGAG
MUNC-18, h/m	GACCTGTCCCAGATGCTGAAGAA, GAGGTGAGCCATGTTGGTGATG
NSF, h/m	GCTCAGTTGTGTGGTTGTGGATGAC, GCTGCTCTCCTGTGGCAATGTT
Rab3A, h	CGTCCTTCCTCTTCCGCTATGCT, TGACACCACCCGCTCATCCTC
Rab3A, m	AAGGTCAAAACCATCTACCGCAACG, CCTCAAAGAACTCAAAGCCCAGGTG
Rab3B, h	GTTCACCCCCAGCCTTCGTTAGCAC, GCCTGCCTTACACTGATGTTCTCCTTT
Rab3B, m	ACCTCCTTCCTTTTCCGCTATGCTG, TGCCTCACACTGATGTTCTCCTTGG
Rabphillin3A, h/m	GGAGCCAGCAAGTCCAACAAGC, CCATGTCCGGTTTCAGCCAGAG
SNAP-23, h	AGAAGATGAAATGGAAGAGAACCTG, CTGTTGGTGTCAGCCTTGTC
SNAP-23, m	GCAAGGGGAACAACTAAATCGCATA, TCCATCTCATCTTCTCTGGCATCATT
SNAP-25, h	GGTATCAGGACTTTGGTTATGTTGGATG, TGGTTTTGTTGGAATCAGCCTTCT
SNAP-25, m	GTTATGTTGGATGAGCAAGGCGAAC, ATCTGGCGATTCTGGGTGTCAAT
Syntaphilin, h/m	GCGCACCTCTCCACCTGTGA, GAGCCGGTCCTGTGTGTCCTT
Syntaxin 1a, h/m	GAGGAGGTGAAGCGGAAGCACAG, AGAGGCAAAGATGGCGGGGTTC
Syntaxin 4, h	CAGGAGTTGGAGAAACAGCAGGTCA, ATCAGACACCATCCCAGCATTGG
Syntaxin 4, m	AGTGGGCAGAGTGAGGTGTTTGTGT, CCAATGATGACAGCCAAGATGAGAA
VAMP1, h/m	ACCCAGGCACAAGTGGAGGAGGT, CAATAACTACCACGATGATGGCACAGA
VAMP3, h	TGGACAAGGTTCTGGAAAGAGACCA, TGAAGAGACAACCCACACGATGATGATG
VAMP3, m	TGGACAAGGTTCTGGAAAGAGACCA, ACACTGATCCCTATCGCCCACATCT
VGLUT1, h	GCCTCCCTCGCCGCTACATTATCG, CGCCGCCAGGGAGTGCTA
VGLUT2, h/m	TGGACATGGTCAACAACAGCA, GCATAGGAACCACAAAAGGAGGT
VGLUT3, h	TGCAATAAGTAAGGTGGGTCTCTTGTC, GCTATGAGGAACACATTCTGCCATT
VGLUT3, m	ACTTTGAGGAGGTCTTTGGATTTGC, TCTCTCTGTTGTCTCCACTCGGTCT
